# Citrus Seed Oils Efficacy against Larvae of *Aedes aegypti*

**Published:** 2017-09-08

**Authors:** Hazrat Bilal, Waseem Akram, Soaib Ali Hassan, Sadrud Din

**Affiliations:** 1 Medical Entomology and Disease Vector Control, Health Services Academy, Islamabad, Pakistan; 2 Department of Agriculture Entomology, University of Agriculture, Faisalabad, Pakistan; 3 Water, Agriculture and Technology Transfer Program, Kabul, Afghanistan

**Keywords:** Citrus cultivars, *Aedes aegypti* larvae

## Abstract

**Background:**

Dengue fever is a serious public health issue in Pakistan for many years. Globally plants have been reported to contain compounds with insecticidal properties. These properties have been demonstrated more recently on the larval stages of mosquitoes. Therefore, Citrus cultivar seeds were evaluated for larvicidal potential against the primary dengue vector *Aedes aegypti*.

**Methods:**

Extraction of oil was done by a steam distillation method and oils were evaluated according to WHO guidelines for larvicides 2005 for evaluation of insecticidal properties of citrus seed extracts against mosquito larvae.

**Result:**

Among the Citrus cultivar seed oil, rough lemon (*Citrus jambhiri*) had the lowest LC_50_ value (200.79ppm), while musambi (*C. sinensis* var *musambi*) had the highest LC_50_ value (457.30ppm) after 24 h of exposure.

**Conclusion:**

Citrus cultivars have some larvicidal potential but *C. jambhiri* had the greatest potential against *A. aegypti* larvae. Further small-scale field trials using the extracts of *C. jambhiri* will be conducted to determine operational feasibility.

## Introduction

Dengue fever (DF) and dengue hemorrhagic fever (DHF) are serious public health concerns in many developing countries, including Pakistan. Over 2.5 billion people over 40% of the world’s population are now at risk from dengue. WHO currently estimates 50–100 million dengue infections occurred worldwide every year (1).

In Pakistan, the first case of DHF was observed in Karachi in 1994, (2) and 11,024 confirmed cases of DF including 40 deaths were reported in 2010 (3). In 2011, a subsequent dengue outbreak occurred with 22,778 confirmed cases and 353 deaths (4). The majority of the cases and deaths occurred in Lahore, Punjab Province while there was also epidemic in Khyber Paktunkhua (KPK) during 2013 that resulted in 23 deaths Apart from the KPK province, sporadic cases have also been reported from the provinces of Punjab, Sindh, and Balochistan (5).

Unlike yellow fever, there is no vaccine for dengue fever. However Osorio et al. (6) is developing a vaccine, which is still in the preclinical stage. Therefore, the only current effective approach to control dengue is through vector control. This is done mainly through integrated vector management programmes using insecticides, environmental management and public awareness (7, 8). Due to the toxic effects and resistance to synthetic insecticides (9), vector control managers are facing problems in controlling mosquitoes. Therefore, it is necessary to develop safe alternative insecticides, which require minimum care (10).

To overcome these problems, concentration has been shifted steadily to the use of biopesticides as adulticides (11), larvicides (12), and repellents (13) providing alternatives to synthetic chemicals. Many investigators have reported on the efficiency of biopesticides (plant extracts) against mosquito larvae (14). Recent studies have encouraged the investigation of insecticidal properties of botanicals. Muthukrishnan and Puspalatha (15) evaluated the larvicidal effects of extracts from *Rhinacanthus nasutus* (Acanthaceae), *Solanum suratense* (Solanaceae), *Calophyllum inophyllum* (Clusiaceae), *Samadera indica* (Simaroubaceae) and *Myriophyllum spicatum* (Haloragaceae) against *Aedes aegypti*, *Culex quinquefasciatus* and *Anopheles stephensi* and concluded that they are environmentally safe, degradable and target specific (16).

In the view of an increased interest in the development of plant-based insecticides as alternatives to synthetic insecticides, this study was conducted to assess the larvicidal potential of citrus cultivars against the dengue vector *A. aegypti.*

## Materials and Methods

### Collection and Rearing of mosquitoes

*Aedes aegypti* larvae were collected from old tire shops of Sargodha (32.0836° N, 72.6711° E) and, reared in 3-inch deep steel trays, and were reared in the insectary of biosystematics Lab University of Agriculture Faisalabad-Pakistan. Larvae were fed on Tetra-min® fish feed until adult emergence (17). Adults were maintained in well-aerated cages (70× 35× 35cm), where the mosquitoes were provided with cotton wicks soaked in 10% sucrose solution. Females were also fed blood from white mice every alternate day (18). A Petri dish with landing pad was provided to lay their eggs. The population was maintained at set conditions of 27±2 °C, 75±5% RH and L14:D10 photoperiod.

### Extraction of oil

The seeds of citrus cultivars were washed to remove the pulp, oven dried for 48 hours at 60 °C and later ground in an electric grinder. A thimble was used to hold the grounded material and kept in an extraction tube of Soxhelt apparatus with extractor ID 38mm, extractor volume 85ml and flask volume 250ml (19) for the extraction of oil by steam distillation method using Diethyl-ether as solvent (250ml/20g sample). The cyclic time of extraction for each sample was 4–5h.

### Bioassay

Six different concentrations increasing by 100ppm from 300–800ppm of extracted oils were used with three replicates for each treatment, each replicate containing 200ml of the oil solution placed in 250ml glass beakers. Batches of 30 late 3^rd^ and early 4^th^ instar larvae of the *A. aegypti* were placed in each beaker (20). Control beakers were treated with diethyl-ether only. The experiment was conducted using Completely Randomized Design (CRD) under lab conditions at 27±2 °C and 70±5% relative humidity.

### Data analysis

Abbot’s formula (21) was used to correct for mortality, and this data was analyzed by probit analysis (22) using Minitab^®^ Statistical Software (23) software to create a dose mortality regression line. In the control treatment, if mortality rates were between 5% to 20% then percent mortality was corrected by Abbot’s formula as follows:

$$\%\;\text{corrected}\;\text{mortality}=\frac{\%\;\text{observed}\;\text{mortality}-\%\;\text{control}\;\text{mortality}}{100-\%\;\text{control}\;\text{mortality}}\times 100$$

## Results

The efficacy of citrus seed oils against the late 3^rd^ and early 4^th^ instar larvae of *A. aegypti* was expressed in terms of LC_50_ as shown in Table 2. Among the oils tested, rough lemon had the lowest LC_50_ value (200.79ppm) which is highly significant (0.001), followed by valencia late (213.02ppm), chakutra (221.40) and narangi (248.16ppm). Musambi had the highest LC_50_ value (457.30ppm) followed by freutrall early (337.63ppm), kinnow (321.60 ppm), succari (316.60ppm) and red blood orange (286.41ppm) after 24h of exposure.

**Table 1.** Following Citrus cultivars were collected from Sargodha

**Sr.#**	**Common Name**	**Botanical Name**
**1**	Chakutra	(*Citrus grandis*)
**2**	Kinnow	(*Citrus reticulate*)
**3**	Musambi	(*Citrus sinensis* var *musambi*)
**4**	Narangi	(*Citrus mitis*)
**5**	Red blood orange	(*Citrus sinensis*)
**6**	Rough lemon	(*Citrus jambhiri*)
**7**	Feutrell	(*Citrus reticulate*)
**8**	Valencia late	(*Citrus sinensis* var *valencia late*)
**9**	Succari	(*Citrus sinensis* var *succari*)

**Table 2.** LC_50_values of citrus seed extract against late 3^rd^ and early 4th instar larvae of *Aedes aegypti* after 24 hours of exposure

**Citrus extracts**	LC50* (ppm)	95% FL (LFL**-UFL***)	**Slope ± S.E**	**χ^2^**	***P***
**Chakutra (*Citrus grandis*)**	221.40	155.29–268.40	1.20±0.23	5.86	0.19
**Kinnow (*Citrus reticulate*)**	321.60	266.89–379.10	1.09±0.15	1.15	0.76
**Musambi (*Citrus sinensis*)**	457.30	412.25–512.18	1.22±0.16	1.10	0.77
**Narangi (*Citrus mitis*)**	248.16	195.98–275.87	1.38± 0.25	6.74	0.12
**Red blood orange (*Citrus sinensis*)**	286.41	262.64–305.54	3.38±0.30	5.44	0.23
**Rough lemon (*Citrus jambhiri*)**	200.79	167.62–230.80	1.75±0.22	10.72	0.001
**Feutrell early (*Citrus reticulate*)**	337.63	298.24–397.30	1.06±0.14	0.55	0.87
**Valencia late (*Citrus sinensis*)**	213.02	138.20–161.42	1.01±0.16	2.20	0.43
**Succri (*Citrus sinensis*)**	316.60	267.00–351.10	1.04±0.14	2.04	0.60

* LC_50_ ie, lethal concentration (ppm) to kill 50% population of the subjected organism

** Lower Fiducial Limit

*** Upper Fiducial Limit

## Discussion

Mosquito borne diseases are one of the most important public health problems in the developing countries like Pakistan. Use of synthetic insecticides are the best option to control mosquito larvae but resistance, environmental problems etc are the some concerned problems which can be overcome by the use of Plant essential oils and extracts as a part of Integrated Vector Management (IVM).

A variety of plants is well known to contain chemicals with bioactive potential (24) as deterrents and attractants (25). The ether oils of different citrus cultivars (Table 2) have been studied as natural insecticides against *A. aegypti* larvae instead of synthetic insecticides as they are reported resistant to the mosquitoes especially *A. albopictus* (26). Rough lemon (*C. jambhiri*) had the lowest LC_50_ value (200.79ppm) against *A. aegypti* larvae. This is well supported by the findings of Akram et al. (17) in *A. albopictus* larvae. Other citrus seed oils also have some larvicidal effect as reported by Hafeez at al. (27) Din et al. (28) against *A. albopictus*. Sumroiphon et al. (29) reported the effects of citrus seed extract against the larvae of *A. aegypti* and *Culex quinquefasciatus*. Extracts of *C. sinensis* (30) and *C. bergamia* (31) have been analyzed for the toxicity against the larvae of *A. aegypti* and found quite effective. Bagavan et al. (32) reported the peel chloroform extract of *C. sinensis*, leaf ethyl acetate extracts of *Ocimum sanctum*, *O. canum* and leaf chloroform extract of *Rhinacanthus nasutus* as possible insecticides against the larvae of *Anopheles subpictus* and methanol extract of *Citrus sinensis* peel, methanol extract of *O. canum* leaves, and ethyl acetate extracts of *R. nasutus* and *O. sanctum* against the larvae of *Culex tritaeniorhynchus*.

## Conclusion

Our results indicated that out of the 9 citrus seed oils, rough lemon (*C. jambhiri*) had good larvicidal potential against late 3^rd^ and early 4^th^ instar larvae of *A. aegypti* in terms of LC_50_. Further studies should be done to investigate the larvicidal potential of rough lemon oil as well as other seed oils for the control of *Aedes* mosquitoes under field conditions.
